# Exploring the membrane potential of a simple dual membrane system by using a constant electric field

**DOI:** 10.1186/1471-2105-16-S8-A5

**Published:** 2015-04-30

**Authors:** Yerko Escalona, Jose A Garate, Tomas Perez-Acle

**Affiliations:** 1 Laboratorio de Biología Computacional (DLab), Fundación Ciencia & Vida, Chile

## Background

Connexins (Cxs) constitute Gap Junction Channels (GJCs). GJCs connect the cytoplasm of adjacent cells providing a hydrophilic path between cells that allow the movement, by passive diffusion, of water, cations and small molecules. The opening or closing of GJCs is dependent on the voltage difference between the apposed cells and/or the membrane potential. An approach to understand the voltage gating mechanisms of GJCs is to study a simplified system that can account for the basic features of a GJC.

## Results

In this work, we have devised a series of simple systems bearing in mind that idea. The systems here presented are: i) a dual membrane, ii) a dual membrane with a pore on each membrane, iii) a dual membrane with a channel connecting both membranes and iv) a dual membrane with a channel having explicit charges inside.

In all cases, membrane and pore were built solely with carbon atoms. Both equilibrium and non-equilibrium MD simulations were performed in all systems. Non-equilibrium simulations were produced by applying a uniform external electric field in order to produce a potential difference across the membranes. We then performed detailed analyses of the electrostatic potential, ionic current and the potential of mean force of an ion through the system pores.

## Conclusions

This study provided important insights regarding the behavior of the electrostatic potential and ion currents inside simple dual membrane systems with or without a connecting channel, and will be useful in understanding the voltage effects and ion transport mechanisms of GJCs.

